# Dr. Paul Segond

**Published:** 1912-12

**Authors:** 


Dr. Paul Segond, Surgeon-in-Chief at the Salpetriere, Paris, died Oct. 27.

